# hnRNP I Inhibits Notch Signaling and Regulates Intestinal Epithelial Homeostasis in the Zebrafish

**DOI:** 10.1371/journal.pgen.1000363

**Published:** 2009-02-06

**Authors:** Jing Yang, Chin Yee Chan, Bo Jiang, Xueyuan Yu, Guo-Zhang Zhu, Yiping Chen, John Barnard, Wenyan Mei

**Affiliations:** 1 Center for Cell and Development Biology, The Research Institute at Nationwide Children's Hospital, Columbus, Ohio, United States of America; 2 Department of Pediatrics, The Ohio State University, Columbus, Ohio, United States of America; 3 Department of Oral Biology, The Ohio State University Health Sciences Center, Columbus, Ohio, United States of America; 4 Department of Biological Sciences, Marshall University, Huntington, West Virginia, United States of America; Harvard Medical School, United States of America

## Abstract

Regulated intestinal stem cell proliferation and differentiation are required for normal intestinal homeostasis and repair after injury. The Notch signaling pathway plays fundamental roles in the intestinal epithelium. Despite the fact that Notch signaling maintains intestinal stem cells in a proliferative state and promotes absorptive cell differentiation in most species, it remains largely unclear how Notch signaling itself is precisely controlled during intestinal homeostasis. We characterized the intestinal phenotypes of *brom bones*, a zebrafish mutant carrying a nonsense mutation in *hnRNP I*. We found that the *brom bones* mutant displays a number of intestinal defects, including compromised secretory goblet cell differentiation, hyperproliferation, and enhanced apoptosis. These phenotypes are accompanied by a markedly elevated Notch signaling activity in the intestinal epithelium. When overexpressed, hnRNP I destabilizes the Notch intracellular domain (NICD) and inhibits Notch signaling. This activity of hnRNP I is conserved from zebrafish to human. In addition, our biochemistry experiments demonstrate that the effect of hnRNP I on NICD turnover requires the C-terminal portion of the RAM domain of NICD. Our results demonstrate that hnRNP I is an evolutionarily conserved Notch inhibitor and plays an essential role in intestinal homeostasis.

## Author Summary

Many gastrointestinal diseases are characterized by unbalanced proliferation and differentiation of intestinal epithelial cells. Accumulating evidence implicates the Notch pathway as a fundamental regulator of intestinal epithelial proliferation and differentiation. Deregulation of Notch causes intestinal defects, such as abnormal intestinal cell lineage development and uncontrolled intestinal cell growth. Thus, a more comprehensive understanding of mechanisms by which the Notch pathway is regulated in intestinal epithelial cells will provide fundamental insights into human intestinal diseases. We report here that mutation in *hnRNP I* elevates Notch signaling in the adult zebrafish intestine and causes abnormal intestinal epithelial cell lineage development and uncontrolled intestinal cell growth. We provide evidence that overexpression of hnRNP I promotes the degradation of Notch intracellular domain (NICD) and inhibits Notch signaling. Our results provide the first evidence that hnRNP I plays critical roles in intestinal homeostasis.

## Introduction

The intestinal epithelium undergoes rapid cell turnover. Renewal of the intestinal epithelium relies on intestinal stem cells in the crypts of Lieberkuhn that are distributed circumferentially around the base of finger-like intestinal villi. New intestinal epithelial cells are continuously produced by stem cells in the crypt and migrate along the crypt-villi axis. During migration, intestinal epithelial cells exit mitotic cell cycle and differentiate. This replaces the cell loss at the tips of villi. Intestinal villi are composed of two differentiated post-mitotic cell lineages: absorptive cells (or enterocytes) and secretory cells, including goblet cells, enteroendocrine cells, and Paneth cells in mammals [1]. Deregulation of intestinal cell proliferation and differentiation impairs the renewal of the intestinal epithelium and causes digestive diseases.

Several signaling pathways are involved in the renewal of the intestinal epithelium [2],[3]. Among these is the Notch pathway, a highly conserved signaling pathway that also regulates many other stem cell lineages during embryonic development and adult tissue homeostasis [3],[4]. Notch signaling is triggered by the interaction between Notch and its ligands Delta/Jagged. Upon ligand binding, Notch undergoes sequential proteolytic cleavages, leading to the release of the Notch intracellular domain (NICD). Subsequently, NICD translocates into the nucleus, where it binds to the transcription factor, CSL (also known as RBP-J in mice, CBF-1 in human, Suppressor of Hairless (Su(H)) in *Drosophila,* LAG-1 in *C. elegans*). This converts CSL from a transcriptional repressor into a transcriptional activator and activates the transcription of Notch target genes [5],[6].

The Notch pathway is active in intestinal stem cells, as judged by the restricted expression of the Notch pathway components and Notch target genes in the crypts [7]–[9]. When Notch signaling is overactivated, it expands the intestinal stem cell population and compromises secretory cell differentiation, without affecting absorptive cell differentiation [10],[11]. Conversely, inhibition of the Notch pathway results in an overproduction of secretory cells at the expense of both stem cells in the crypts and absorptive cells [9],[12]. Consistently, ablation of Notch target genes impairs intestinal epithelial homeostasis [7],[13],[14]. It is widely believed that Notch signaling maintains intestinal stem cells in a proliferative state and promotes the absorptive cell fate determination in vertebrate intestine. However, it remains largely unclear how Notch signaling is precisely regulated in the intestinal epithelium.

Heterogeneous nuclear ribonucleoprotein (hnRNP) family RNA binding proteins have been implicated in various aspects of RNA metabolism in a range of biological processes [15]. Among these is hnRNP I (also known as polypyrimidine tract-binding protein, PTB), which regulates tissue specific mRNA alternative splicing [16], mRNA stability [17], localization[18], and translation [19]. Interfering with hnRNP I impairs *Xenopus* skin development [20], *Drosophila* spermatogenesis [21], and *Drosophila* wing development [22]. Like many hnRNP family members, hnRNP I is expressed in the intestine [23]. Yet the function of hnRNP I in the intestine has not been reported. Here we provide the evidence that hnRNP I is an evolutionarily conserved Notch inhibitor and plays a critical role in the intestinal epithelial cell lineage development.

## Results

### The Abnormal Intestinal Epithelium Architecture in *brom bones*

hnRNP I RNA binding protein is composed of four RNA-recognition motifs (RRMs), a nuclear localization signal, and a nuclear export signal [24],[25]. All four RRMs are involved in RNA binding [26]–[31]. We have identified a zebrafish mutant *brom bones* [32], which carries a nonsense mutation in *hnRNP I* gene. The mutation occurs in the middle of the second RRM (Wenyan Mei and Mary C. Mullins, unpublished data). The truncated protein lacks 60% of amino acid residues and only contains the nuclear localization/export signals, the first RRM, and the N-terminal portion of the second RRM. As expected, the RNA binding activity of the mutant protein is severely reduced (data not shown).

**Figure 1:**
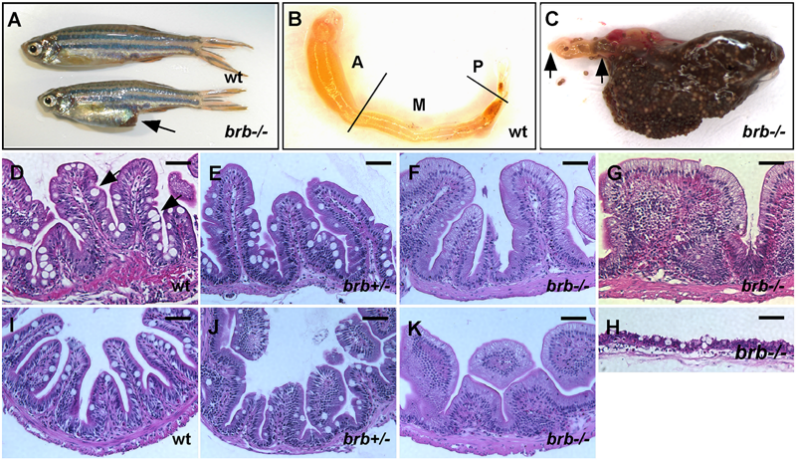
The abnormal intestinal architecture in *brom bones*. (A) *brom bones* displays an enlarged abdomen (arrow) as compared to a wild-type sibling. (B) The dissected intestine of the wild-type fish shown in (A). The original coiled intestine tube was unfolded for a better view. Lines indicate the borders between anterior, mid and posterior intestines. (C) The intestine dissected from the *brom bones* mutant shown in (A). Note the abundant food waste in the intestine. Double arrows indicate the clear portion in the anterior intestine. Anterior is toward left. (D–K) Hematoxylin and Eosin (H&E) staining on cross-sections of the intestines from a wild-type fish (D and I), a *brom bones* heterozygous fish (E,J), and *brom bones* homozygous fish (F, G, H, and K). (D–H) From the anterior segments of the intestine. (I–K) From the mid segments. Arrows in (D) point to goblet cells. The severity of the intestinal phenotype varies among individual homozygous mutants. (F) represents *brom bones* homozygous intestines that display markedly reduced goblet cells. (G) represents *brom bones* homozygous intestines that exhibit excessive number of the intestinal epithelial cells and lack visible lamina propria. (H) is the cross-section of the *brom bones* intestine shown in (C), representing mutants with the big abdomen phenotype. Note that both the intestinal epithelium and the smooth muscle layer undergo degeneration. *brb* = *brom bones*; wt = wild-type; A = anterior intestine; M = mid intestine; P = posterior intestine. Scale bar = 50 µm.

Homozygous *brom bones* mutants (hereafter referred to as *brom bones*) are viable. However, a fraction (9 out of 27) of aged *brom bones* fish (>9 months) showed bigger abdomens (Figure 1A, arrow) when compared to their age-matched heterozygous and wild-type sibling fish (0 out of 89). We dissected the intestine from a *brom bones* homozygous fish with the big abdomen and examined its anatomy. As shown in Figure 1B, the intestine from a wild-type adult fish has a tube-like shape and can be divided into anterior, mid and posterior segments based on the height of the intestinal fold and the distribution of differentiated intestinal epithelial cell types [33]. Sparse fecal material can be found occasionally in all segments of the intestinal tubes in wild-type fish (not shown). In striking contrast, the intestine of the *brom bones* mutant with the “big abdomen” phenotype is full of fecal material (Figure 1C). The food wastes can be found in all three intestinal segments, with the exception of the very anterior portion of the intestinal bulb (Figure 1C, double arrows). It appears that *brom bones* mutants have difficulties in compacting food wastes into feces and expelling it through the anus. These affected *brom bones* fish usually die shortly after the appearance of the big abdomen phenotype. Interestingly, we have noticed that the big abdomen phenotype is genetic background-dependent. The phenotype is very severe on the AB and Tubingen background, but less prominent on the WIK background.

To characterize phenotypic defects in the *brom bones* intestine, we carried out histological analysis. As shown in Figure 1D, the organization of the wild-type adult fish intestine is very similar to that of neonatal mammals. Villi of the intestinal epithelium are organized into ordered periodic protrusions, which vary in width and lack crypts of Lieberkuhn (Figure 1D and 1I and [12],[33],[34]). A number of goblet-like cells, characteristic of large apical mucin filled area, can be easily identified along the villous epithelium (pointed by arrows in Figure 1D). In addition, a muscular layer lies immediately beneath the base of villi. Little if any abnormalities can be detected in the intestinal epithelium of *brom bones* heterozygous fish (Figure 1E and 1J). *brom bones* homozygous mutants, however, display abnormal intestinal epithelium. The most severe phenotype was observed in mutants with the big abdomen phenotype. In these fish, both the intestinal epithelium and the underlying smooth muscle layer undergo severe degeneration (Figure 1H). We also analyzed homozygous mutants lacking the big abdomen phenotype. While 17% of mutants (4 out of 23) display normal intestinal epithelium architectures (not shown), 83% of mutants (19 out of 23) show a remarkable decrease in the number of goblet-like cells in both the anterior and the mid segments of their intestines (Figure 1F, 1G, and 1K). In addition to the decrease in the number of goblet-like cells, 17% of mutants (4 out of 23) display a more severe phenotype. The intestinal villi of these fish appear very wide and contain an excessive number of intestinal epithelial cells (Figure 1G). Lamina propria, which separates the epithelium from the underlying smooth muscle layers, is not visible. In contrast to the remarkable decrease in the number of goblet-like cells, the columnar-shaped enterocytes in *brom bones* mutants are indistinguishable from those in the wild-type intestine. These results suggest that *hnRNP I*, which is mutated in *brom bones,* is required for maintaining a normal intestinal architecture in adult zebrafish.

Because the phenotypes in the anterior and the mid segments of the *brom bones* intestine are very similar, we chose the anterior segment of the intestine for detailed analysis.

### Impaired Goblet Cell Differentiation in the *brom bones* Intestinal Epithelium

The above histological analysis indicates that the number of goblet-like cells is reduced in the *brom bones* intestine, suggesting that cell fate determination may be altered in the *brom bones* intestinal epithelium. To determine whether this is the case, we examined the expression of goblet cell and enterocyte markers in the control and *brom bones* intestines.

**Figure 2:**
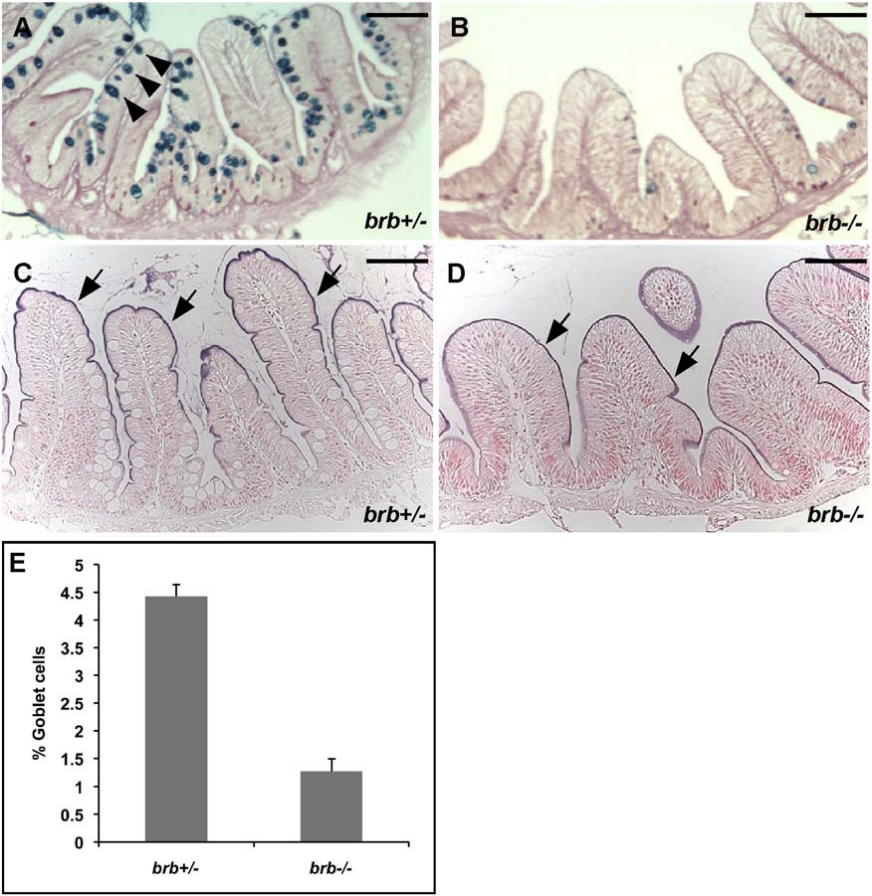
*brom bones* has significantly reduced number of intestinal goblet cells. (A,B) PAS-AB staining shows the intestinal goblet cells (arrowheads in A) in the anterior intestines of *brom bones* heterozygous fish (A) and homozygous fish (B). (C,D) The enzymatic activity of intestinal alkaline phosphatase (AP) in the enterocytes brush border (arrows) of the anterior intestines in *brom bones* heterozygous fish (C) and homozygous fish (D). (E) Quantification of the goblet cell number. Values shown are the percentages of goblet cells relative to the total number of intestinal epithelial cells per villous cross-section (n = 129 villi from 7 *brom bones* heterozygous fish and 154 villi from 9 *brom bones* homozygous fish). Error bars represent s.e.m; *P* = 1×10^−6^ by Student's t test. Scale bar = 50 µm.

The function of intestinal goblet cells is to secrete mucus. To identify goblet cells, we performed Alcian blue-periodic acid Schiff (AB-PAS) histochemical staining, a method specific for detecting mucin [35]. Aged-matched *brom bones* heterozygous intestines were used as controls (Figure 2A). In the control intestines, 4.4% of intestinal epithelial cells are goblet cells (n = 7 *brom bones* heterozygous fish) (Figure 2A and 2E). In contrast, only 1.3% of cells in the *brom bones* intestinal epithelium are goblet cells (n = 9 *brom bones* homozygous fish) (Figure 2B and 2E). Intestinal alkaline phosphatase (AP) is a specific marker for the brush border of enterocytes [36],[37]. We thus examined the enzymatic activity of AP to identify enterocytes. As pointed by the arrows in Figure 2C and D, the enzymatic activity of AP in the intestinal epithelium of *brom bones* (100%, n = 20 *brom bones* homozygous fish) was indistinguishable from that in controls. We conclude that *hnRNP I* is required for intestinal goblet cell differentiation.

### Increased Cell Proliferation and Apoptosis in the *brom bones* Intestinal Epithelium

Altered intestinal lineage development is often accompanied by modification in cell proliferation or cell survival. To determine whether cell proliferation and cell survival are altered in the *brom bones* intestine, we examined the expression of a cell proliferation marker, proliferating cell nuclear antigen (PCNA) [12],^34^ and a cell apoptosis marker, active caspase 3 [9].

**Figure 3:**
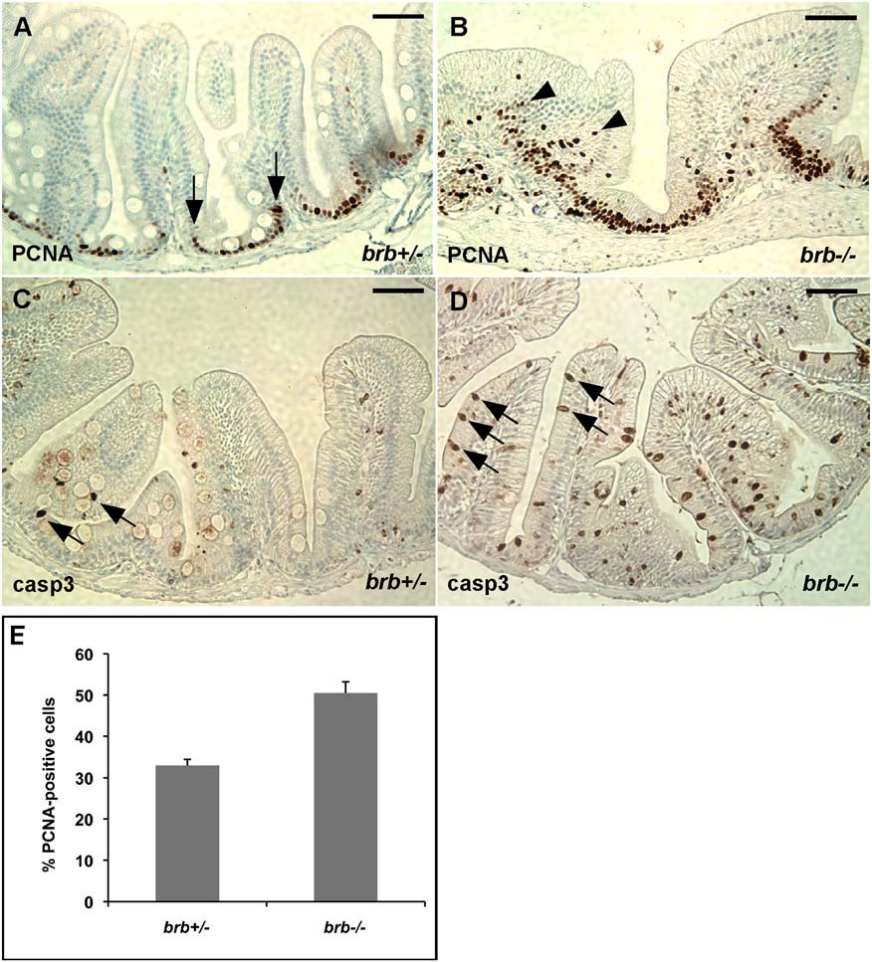
*brom bones* has increased cell proliferation and apoptosis in the intestinal epithelial cells. (A,B) Representative cross-sections of the anterior intestines from control and mutant fish immunostained for PCNA. Arrows in (A) mark the border of PCNA expression region in the intervillus pocket of a control fish. Arrowheads in (B) indicate PCNA-positive cells in the center region of intestinal epithelium of a *brom bones* homozygous fish. (C,D) Representative cross-sections of the anterior intestines from control (C) and mutant (D) fish immunostained for active caspase 3. Arrows point to caspase-positive cells. (E) Quantification of the PCNA-positive cell number. Values shown are the percentages of PCNA-positive cells relative to the total number of intestinal epithelial cells per villous cross-section (n = 97 villi from 6 *brom bones* heterozygous fish and 98 villi from 8 *brom bones* homozygous fish). Error bars represent s.e.m; *P* = 2×10^−4^ by Student's t test. Scale bar = 50 µm.

In the control intestine, cells positive for PCNA staining are mainly located in the intervillus pocket (Figure 3A, double arrows), which is functionally equivalent to the crypt of the mammalian intestine [12],[34],[35]. In contrast, PCNA-positive cells are not only located in the intervillus pockets, but also extended distally onto the intestinal villus in *brom bones* (Figure 3B, arrowheads). The percentage of PCNA-positive cells relative to the total intestinal epithelial cells is markedly increased in the *brom bones* homozygous intestine (50.5%, n = 8 *brom bones* homozygous fish) when compared to that in controls (33.0%, n = 6 *brom bones* heterozygous fish) (Figure 3E). In addition to enhanced cell proliferation, most *brom bones* intestines (75%, n = 12 *brom bones* homozygous fish) exhibit a significant increase in the number of caspase 3-positive cells when compared to the control intestines (Figure 3C and 3D). Thus, loss of *hnRNP I* enhances cell proliferation and apoptosis in the zebrafish intestinal epithelium.

### Elevated Notch Signaling in the *brom bones* Intestinal Epithelium

The abnormalities observed in the *brom bones* intestine, including a decrease in the number of goblet cells, an increase in the levels of cell proliferation and cell apoptosis, resemble the remarkable intestinal phenotypes observed in mice with elevated Notch signaling [10],[11]. Interestingly, Dansereau et al reported that hnRNP I inhibits Notch signaling during *Drosophila* wing development [22]. Thus, we went to determine whether Notch signaling is elevated in the *brom bones* intestine.

**Figure 4:**
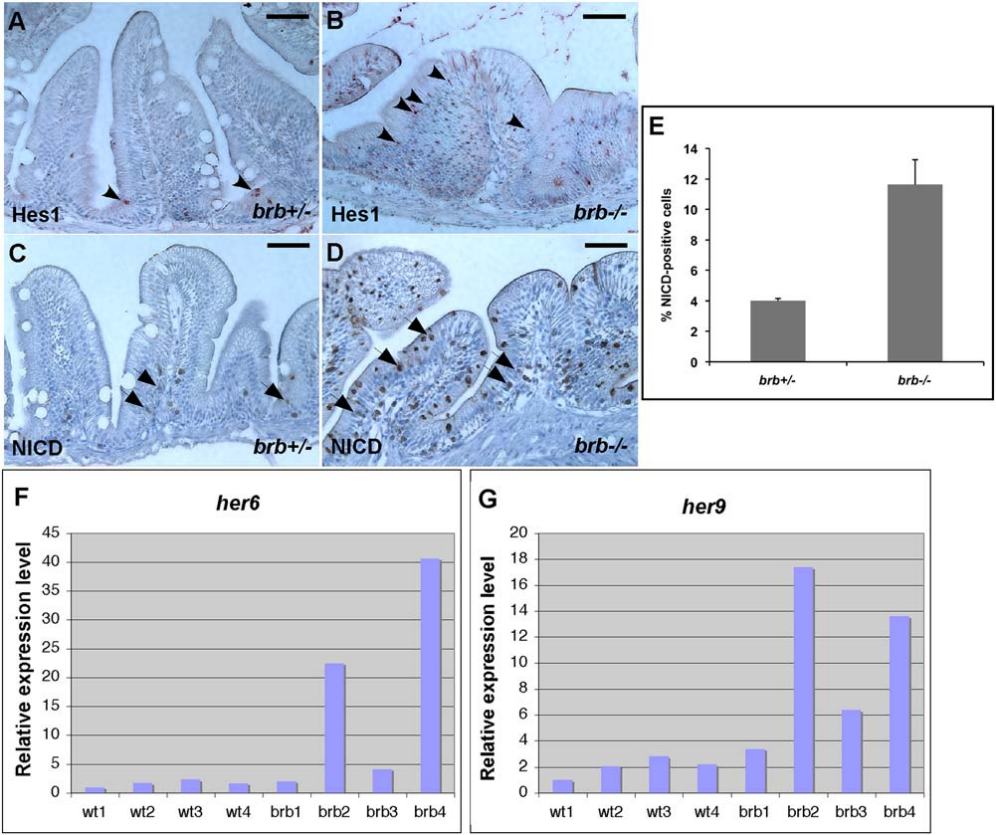
*brom bones* possesses an increased level of Notch signaling activity in the intestinal epithelium. (A–D) Cross-sections of the anterior intestines from control (A,C) and mutant fish (B,D) immunostained for Hes1 (A,B) and NICD (C,D). Arrowheads in A and B point to Hes1 positive-cells. Arrows in (C) and (D) indicate NICD-positive cells. (E) Quantification of the NICD-positive cell number. Values shown are the percentages of NICD-positive cells relative to the total number of intestinal epithelial cells per villous cross-section (n = 114 villi from 7 *brom bones* heterozygous fish and 175 villi from 10 *brom bones* homozygous fish). Error bars represent s.e.m; *P* = 0.001 by Student's t test. (F,G) Real-time RT-PCR results show the expression levels of *her6* (F) and *her9* (G) in the intestines of 4 wild-type fish and 4 *brom bones* homozygotes. The expression levels of *her6* and *her9* were normalized to *odc*. Gene expression levels in the wild-type 1 fish were set as 100%. Scale bar = 50 µm.

First, the expression of Hes1, a direct target of Notch signaling, was examined by immunostaining. A small number of Hes1-positive cells were detected in the intervillus pocket in the control intestinal epithelium (Figure 4A, arrowheads). The number of Hes1-positive cells in the *brom bones* intestine, however, is dramatically increased. These Hes1-positive cells are located not only in the intervillus pocket, but also distally onto the intestinal villus in *brom bones* mutants (Figure 4B, arrowheads). This phenotype was observed in the majority of *brom bones* mutants analyzed (83%, n = 12 mutant fish). We also examined the expression of *her6* and *her9*, two zebrafish homologs of mammalian *hes1* [38], in the intestine by real-time PCR. Four controls and four *brom bones* intestines were analyzed. Consistent with the partially penetrant intestinal phenotypes described above, the expression of *her6* and *her9* is dramatically increased in two mutants, moderately increased in one mutant, and remains relatively normal in one mutant (Figure 4F and 4G). Thus, mutation in *hnRNP I* results in an increase in Notch target gene expression in the intestinal epithelium.

Next, we examined the level of NICD in the *brom bones* intestinal epithelium using an antibody specific for the active form of Notch [39]–[42]. In the control intestine, 4.0% of intestinal epithelial cells were positive for NICD staining (n = 7 *brom bones* heterozygous fish, Figure 4E). These NICD-positive cells were mainly restricted in the intervillus pocket (Figure 4C, arrows). Intriguingly, the percentage of NICD-positive cells was dramatically increased in the *brom bones* intestine (11.7%, n = 10 *brom bones* homozygous fish, Figure 4E). These NICD-positive cells were detected not only in the intervillus pocket, but also more distally on the villus (Figure 4D, arrows), resembling the distribution of Hes1-positive cells in the mutant intestine. Indeed, using double staining, we found that NICD staining completely overlaps with Hes1 staining (Figure S1), suggesting that the increased Notch target gene expression in the *brom bones* intestine was triggered by the excessive NICD. Taken together, we conclude that Notch signaling activity is elevated in the *brom bones* intestinal epithelium.

### hnRNP I Reduces the Level of Overexpressed NICD

*brom bones* carries a nonsense mutation in *hnRNP I* gene, leading to a big truncation of the hnRNP I protein. The observation that Notch signaling is enhanced in the *brom bones* intestine strongly suggests that, like in *Drosophila* [22], *hnRNP I* inhibits the Notch signaling in vertebrates. To further test this possibility, we investigated the function of *hnRNP I* in *Xenopus* embryos, a vertebrate model widely used for studying Notch signaling.

**Figure 5:**
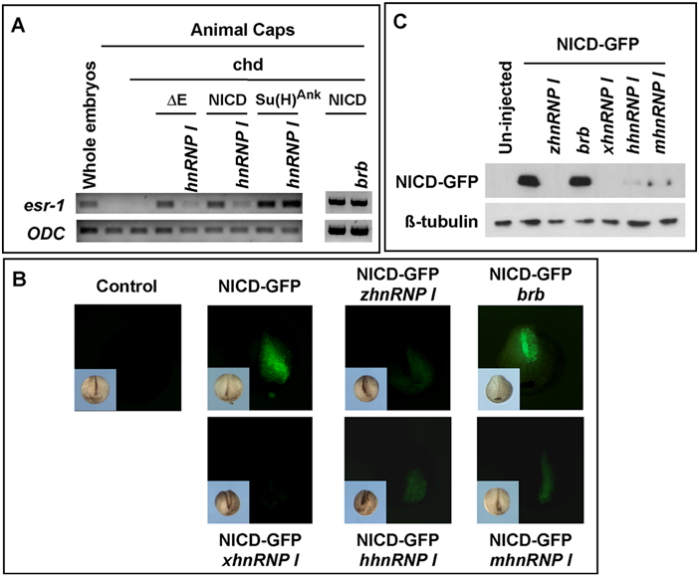
*hnRNP I* inhibits Notch signaling. (A) RT-PCR results demonstrate that *hnRNP I* (1 ng), but not *brb* (1 ng), inhibits the expression of *esr-1* induced by NotchΔE, NICD, but not that induced by Su(H)^Ank^ in *Xenopus* animal caps. Animal caps were neuralized by *chordin* (Chd, 50 pg). (B) Stereoimages show *hnRNP I*s from the wild-type zebrafish (*zhnRNP I,* 2.5 ng), human (*hhnRNP I,* 2.5 ng), mouse (*mhnRNP I,* 2.5 ng) and *Xenopus* (*xhnRNP I,* 2.5 ng) decreased the level of NICD-GFP as revealed by the presence of green fluorescence. In contrast, the mutated form of *hnRNP I* in *brom bones* (*brb*) failed to reduce the level of NICD-GFP. Note that only one of the dorsal animal blastomeres, which later gave rise to the neural tissue, was injected. Inserts at the lower left corner of each panel are bright field images. (C) Western blot result showing the level of NICD-GFP (upper panel) was reduced by overexpression of *hnRNP I*s. Protein extracts were made from 15 injected embryos (20 ml lysis buffer per embryo). Each lane contains 10 ml lysate. After Western blot with the anti-GFP antibody, the membrane was re-probed with anti-tubulin antibody (lower panel).

Ectopic activation of the Notch pathway in neuralized *Xenopus* animal caps induces the expression of *esr1*, a *Xenopus* homolog of mammalian *hes1/5* [43]. We took advantage of this assay and performed an epistasis analysis. To initiate signaling from different levels of the Notch pathway, we overexpressed NotchΔE (2 ng), NICD (1 ng), and Su(H)^Ank^ (0.5 ng). NotchΔE lacks the extracellular domain of Notch. It can be converted into NICD and activate Notch target genes in the presence of an active g-secretase [44]. Su(H)^Ank^ is a constitutively active form of Su(H), which functions independent of NICD [43]. As expected, overexpression of NotchΔE, or NICD, or Su(H)^Ank^ induced *esr1* expression in neuralized animal caps. Co-expression of zebrafish *hnRNP I* (1 ng) reduced the expression *esr1* induced by NotchΔE and NICD, but not that by Su(H)^Ank^ (Figure 5A). In contrast, co-expression of *brb* (1 ng), the mutated form of *hnRNP I* in *brom bones*, failed to block the expression of *esr1* induced by NICD in *Xenopus* animal caps (Figure 5A). This demonstrates that *hnRNP I* functions negatively in the Notch pathway upstream of Su(H).

To further understand the mechanism through which hnRNP I inhibits the Notch pathway, we asked whether overexpression of *hnRNP I* alters the level of NICD. A GFP-tagged NICD (NICD-GFP, 1 ng) was injected into *Xenopus* embryos alone, or together with zebrafish *hnRNP I* (*zhnRNP I* ) (1 ng). As a control, we also co-injected NICD-GFP with *brb* (1 ng). When embryos reached the tailbud stage, green fluorescence signals, which represent the expression of NICD-GFP protein, were detected in embryos injected with NICD-GFP or NICD-GFP/*brb* (Figure 5B). Embryos injected with NICD-GFP/*hnRNP I* either lacked green fluorescence completely (Figure 5B), or only exhibited a very weak level of green fluorescence (not shown). In fact, the level of NICD-GFP was reduced by *hnRNP I*s from other vertebrates as well, including human (*hhnRNP I*, 1 ng), mouse (*mhnRNP I*, 1 ng), and *Xenopus* (*xhnRNP I*, 1 ng) (Figure 5B). To confirm this observation, we prepared protein extracts from injected embryos and performed western blot using an anti-GFP antibody. As shown in Figure 5C, overexpression of *hnRNP I*, but not *brb*, reduced the level of NICD-GFP. These results, therefore, demonstrate that hnRNP I is an evolutionarily conserved inhibitor of the Notch pathway.

### hnRNP I Promotes NICD Turnover through Its Effect on the C-Terminal Portion of the RAM Domain of NICD

**Figure 6:**
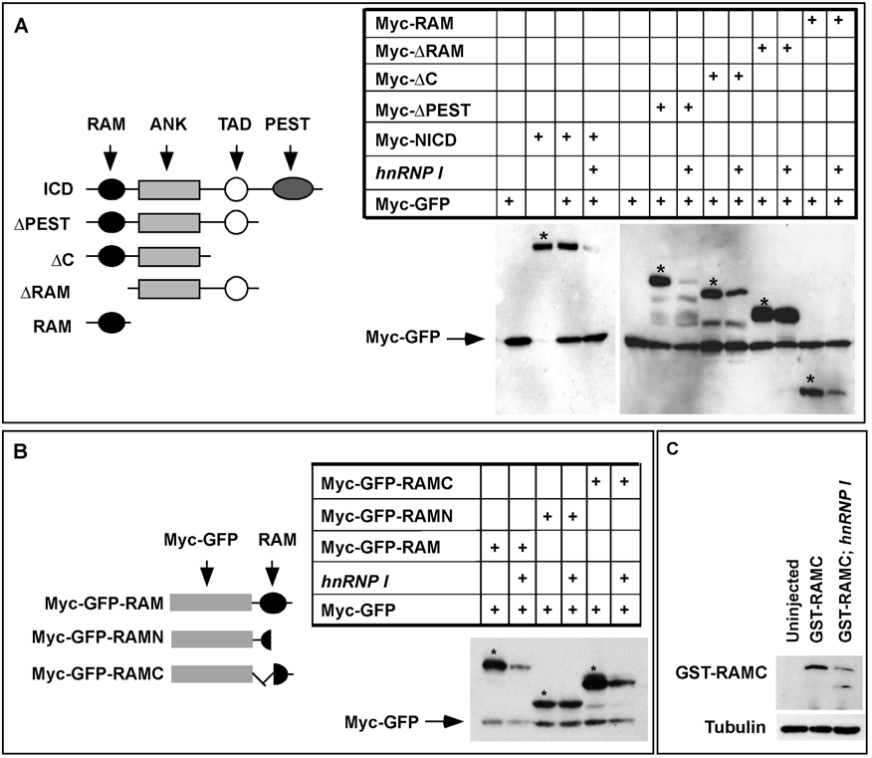
C-terminal region of RAM domain mediates the *hnRNP I*-induced NICD turnover. (A) Schematic representation of serial deletion NICD constructs (left panel) and the Western blot results (right panel) showing that the RAM domain is required for *hnRNP I*-induced NICD turnover. Myc-GFP (pointed by arrow) was used as the control for microinjection and loading. * indicates NICD and NICD deletion constructs. (B) Schematic representation of RAM deletion constructs (left panel) and the Western blot results (right panel) demonstrating that the C-terminal region of RAM domain is required for *hnRNP I*-induced NICD turnover. Myc-GFP was used as the control for injection and loading (pointed by arrow). * indicates RAM and RAM deletion constructs. (C) Western blot result showing that overexpression of *hnRNP I* destabilized GST-RAMC protein purified from bacteria. GST-RAMC protein (250 pg per embryo) was injected alone, or together with *hnRNP I* RNA (2 ng) into *Xenopus* embryos. Control and injected embryos were harvested at the tailbud stage. A total volume of 1 ml lysate, which was prepared from 50 embryos, was incubated with 50 ml glutathione-agarose beads at room temperature for 1 hour to recover GST-RAMC protein. Protein was eluted from the beads and blotted with an anti-GST antibody (upper panel). Supernatants were probed with the anti-tubulin antibody (lower panel).

NICD contains several domains, including a RAM domain, six cdc10/Ankyrin repeats, a transcriptional transactivation domain (TAD), and a C-terminal PEST domain [5]. To further understand the mechanism through which hnRNP I inhibits Notch signaling, we went to determine which domain of NICD mediates the inhibitory effect of hnRNP I on NICD. Several deletion constructs, including ΔPEST, ΔC, ΔRAM, and RAM were generated (Figure 6A). When expressed in *Xenopus* embryos, the expression levels of NICD, ΔPEST, ΔC, and RAM were significantly decreased by the co-expression of *hnRNP I* (Figure 6A). Notably, all these constructs contain the RAM domain. In contrast, the level of ΔRAM, which lacks the RAM domain, was not sensitive to *hnRNP I* overexpression (Figure 6A). This indicates that the RAM domain mediates the inhibitory effect of hnRNP I on NICD.

To further map the hnRNP I responsive motif within the RAM domain, we generated Myc-GFP-RAM, myc-GFP-RAMN, and myc-GFP-RAMC, which contain the entire RAM, or the N-terminal, or the C-terminal region of RAM, respectively (Figure 6B). When expressed in *Xenopus* embryos, the expression levels of myc-GFP-RAM and myc-GFP-RAMC were decreased by *hnRNP I*, whereas overexpression of *hnRNP I* had no effect on the level of myc-GFP-RAMN (Figure 6B). Thus, the C-terminal portion of the RAM domain mediates the inhibitory effect of hnRNP I on NICD.

The above results raise the possibility that hnRNP I promotes the turnover of NICD protein. To determine whether this is the case, we took advantage of RAMC, the minimal motif that mediates the inhibitory effect of hnRNP I on NICD. We first expressed and purified a GST-tagged RAMC protein (GST-RAMC) from bacteria. This GST-RAMC protein was injected into *Xenopus* embryos either alone, or together with *hnRNP I* RNA (2 ng). Injected embryos were harvested at the tailbud stage and the levels of GST-RAMC were determined by Western blot. As shown in Figure 6C, overexpression of *hnRNP I*, indeed, decreased the level of GST-RAMC protein in embryos. This demonstrates that hnRNP I promotes the turnover of RAMC and indicates that hnRNP I destabilizes NICD protein.

## Discussion

Recent studies have highlighted the fundamental roles of Notch signaling in many stem cell lineages [4]. In the intestinal epithelium, the Notch cascade is critical for the proliferation of intestinal stem cells and promotes absorptive cell differentiation. Ectopic activation of Notch signaling enhances proliferation of intestinal stem cells [10],[11], and impairs secretory cell lineage development, without preventing absorptive cell differentiation [9]–[12],[45]. Despite the emerging role of Notch signaling in intestinal homeostasis, it is not clear how Notch signaling is precisely controlled during this process.

Here, we report that hnRNP I is an essential inhibitor of Notch signaling and plays critical roles in the intestinal epithelium. We show that *brom bones,* which is deficient in *hnRNP I*, displays intestinal defects strikingly similar to phenotypes observed in mice with elevated Notch signaling. Indeed, in the *brom bones* intestine, the amount of NICD, a hallmark of activated Notch signaling, is dramatically increased. This is accompanied by markedly enhanced Notch target gene expression. NICD/Hes1-positive cells, which are located in the intervillus pocket in wild-type intestine, were detected more distally on villi in the *brom bones* intestine. This suggests that under physiological conditions, hnRNP I is responsible for turning off Notch signaling when newly derived intestinal epithelial cells migrate along the crypt-villus axis. In agreement with this hypothesis, overexpression of *hnRNP I* inhibits *esr1* expression induced by NotchΔE and NICD in *Xenopus* animal caps. Furthermore, we show that *hnRNP I* from human, mice, *Xenopus* and zebrafish are all capable of promoting NICD turnover. Interestingly, a previous study in *Drosophila* has indicated that loss of *Hephaestus*/*hnRNP I* resulted in stabilization of liberated NICD without affecting the full length Notch in the wing disc [22]. It appears that hnRNP I is an evolutionarily conserved inhibitor of the Notch pathway.

The Notch pathway is essential for embryonic development. Why does mutation in *hnRNP I*, a negative regulator of Notch signaling, fail to affect embryogenesis in *brom bones*? Searching the zebrafish genome reveals the existence of two closely related *hnRNP I* genes. The first one is located on chromosome 2 and is mutated in *brom bones.* The second one is located on chromosome 11. These two hnRNP I proteins share 84% identity and likely function redundantly. Both *hnRNP I* genes are expressed during early embryonic development and in all adult tissues analyzed (our unpublished data). Thus, it is reasonable to consider *brom bones* as a partial loss-of-function mutant. It is likely that in *brom bones* mutants, the total activity of hnRNP I falls below a critical threshold essential for the intestinal homeostasis but not for the embryogenesis. As a consequence, Notch signaling becomes up-regulated in intestinal epithelial cells and the intestinal homeostasis is disrupted.

Interestingly, a recent report showed that depletion of *hnRNP I* by morpholino injection induced skin defects during *Xenopus* embryonic development [20]. Given the important roles of Notch signaling in the skin development [46], it is tempting to speculate that hnRNP I inhibits Notch signaling in this developmental process as well. Nevertheless, further studies are needed to determine if hnRNP I-dependent NICD turnover has a broad impact on embryonic development and adult tissue homeostasis, or whether its function is restricted to only a few lineages.

Mechanistically, how does hnRNP I regulate NICD turnover? It has been reported that interaction between NICD and its co-activators Mastermind and Ski-interacting protein, promotes the phosphorylation of NICD by cyclin-dependent kinase-8 (CDK8). Phosphorylated NICD in turn interacts with the SEL10 E3 ubiquitin ligase through its C-terminal PEST domain, leading to the ubiquitination and proteasome-dependent degradation of NICD [47]–[49]. In addition, the Itch/NEDD4/Su(dx) family of HECT domain E3 ligases can ubiquitinate and target NICD for proteasome-dependent degradation through the RAM-Ankyrin repeat region of the NICD [50]. In *Xenopus* embryos, NRARP (Notch regulated akyrin repeat protein) can form a complex with NICD/Su(H) and promote NICD degradation as well. In this case, NRARP interacts with the Ankyrin repeats of NICD [51]. Results from our deletion assay demonstrate that RAMC, a small motif located in the C-terminus of the RAM domain, mediates hnRNP I-induced NICD turnover. Since the PEST domain and the Ankyrin repeats are not required for hnRNP I-induced NICD turnover, it is unlikely that hnRNP I promotes NICD turnover through the CDK8/SEL10 pathway or NRARP. Currently, it remains unclear if hnRNP I induces NICD degradation through the Itch/NEDD4/Su(dx) cascade, or whether hnRNP I promotes NICD turnover through a novel mechanism. Nevertheless, results presented here allow us to propose a working model. We hypothesize that through its effect on a yet unknown RNA(s), hnRNP I regulates the interaction between NICD and a RAMC binding factor, and promotes the degradation of NICD. This RAMC binding factor itself could be a direct target of hnRNP I. Alternatively, hnRNP I may regulate the production or activity of this RAMC binding factor through an indirect mechanism. It is of interest to identify this RAMC binding factor and investigate the mechanism through which hnRNP I turns off the Notch pathway.

In summary, we show for the first time that the hnRNP-dependent NICD turnover is an evolutionarily conserved inhibitory mechanism for turning off the Notch pathway. Our work demonstrates a novel function of *hnRNP I* in intestinal epithelial homeostasis by regulation of cell proliferation and lineage development.

## Materials and Methods

### Animals

The use of animals in this research was approved by the Research Institute at Nationwide Children's Hospital animal care and use committee (protocol #02904AR for zebrafish and 04104AR for *Xenopus*). Zebrafish breeding were done as described previously [52],[53]. The *brom bones* mutant [32] was maintained in the AB and Tubingen backgrounds or AB and Tubingen and WIK background. For genotyping, genomic DNA was isolated from tail fin [54] and amplified with primers 5′-GCTTAACATTAAACAGTCTTTAGATCGA-3′ and 5′-CTTATCATTGTTGTACTTAACATTCAGG-3′. PCR products were further digested with Cla I to detect a restriction fragment length polymorphism generated by the *brom bones* mutation. All mutant samples shown in this paper were randomly collected from *brom bones* mutants that lack the big abdomen phenotype, with the exception of Figure 1C and 1H, which were chosen to show the intestinal morphology of a mutant with the big abdomen phenotype.

*Xenopus* embryos were obtained as described [55]. Microinjection and animal cap assays were performed as described [56]. The dosage of RNA for microinjection is indicated in the text or figure legends.

### Histology, Immunostaining, Alcian Blue-Periodic Acid Schiff (AB-PAS) Histochemical Staining, and Alkaline Phosphatase (AP) Assay

The intestines were isolated, fixed, paraffin-embedded, and sectioned according to standard protocols. Intestine sections (5–14 µm) were processed for Hematoxylin and Eosin staining or for immunostaining. Immunohistochemistry was performed with R.T.U. vectastain kit (Vector Laboratories) with DAB substrate or AEC substrate. In some experiments, sections were counterstained lightly with Hematoxylin afterwards. Primary antibodies are: mouse anti-PCNA (Sigma, P8825), rabbit anti-active caspase3 (Sigma, C8487), rabbit anti-cleaved Notch1 (Cell signaling, 2421S), and goat anti-Hes1 (Santa Cruz, sc-13842). Secondary antibodies for immunofluorescence are goat anti-rabbit AlexaFluor 488 or 594 and donkey anti-goat AlexaFluor 594 (Invitrogen). For Hes1 and NICD double immunostaining, sections were incubated with anti-Hes1 and anti-NICD antibodies first, then wash with PBS, followed by incubation with the secondary antibody donkey anti-goat AlexaFluor 594. After thoroughly PBS washes, sections were then incubated with the secondary antibody goat anti-rabbit AlexaFluor 488. Goblet cell secreted mucins were identified by sequentially incubating deparaffinized sections in pH 2.5 alcian blue (1 hour), periodic acid (7 minutes) and Schiff's reagent (10 minutes). After the staining, acidic mucins are stained “blue” and neutral mucins are stained red. The enzymatic activity of intestinal AP at enterocytes-brush border was detected with bromochloroindoyl phosphate/nitro blue tetrazolium. Sections were counterstained lightly with Nuclear Fast Red. Images were taken from a dissection or a Compound microscope (Leica) with digital camera or a Zeiss LSM510 confocal microscope and processed using Adobe Photoshop.

### Plasmids, RNA, and GST-Tagged RAMC Protein

NotchΔE, NICD [44],[57], and Su(H)^Ank^ [43] were described. NICD-GFP and Notch deletion constructs were generated by standard cloning strategies. ΔC (V1744-T2128), ΔRAM (C1861-T2128), RAM (V1744-C1861), RAMN (V1744-G1793), and RAMC (N1789-C1861) were PCR amplified from mouse Notch1 and cloned into pCS2-MT or pCS2-MT-EGFP. pCS2-*zhnRNP I* and pCS2-*brb* were generated by RT-PCR, using cDNAs derived from wild-type and *brom bones* fish. A pGEX6P1-RAMC was constructed for expressing the GST-RAMC protein in bacteria. A pCS2-*xhnRNP I* containing the full length *hnRNP I* was obtained by screening a *Xenopus* oocyte cDNA library. Mouse *hnRNP I* (IMAGE: 30439895) and human *hnRNP I* (IMAGE: 3863892) were purchased from ATCC.

RNAs for microinjection were synthesized using the mMESSAGE mMACHINE kit (Ambion). Notch deletion constructs were linearized with AseI and transcribed with SP6 RNA polymerase. *hnRNP I* constructs were linearized with Not I and transcribed with SP6 RNA polymerase.

We followed the standard protocol to express and purify GST-RAMC protein. Briefly, BL21 bacteria containing the pGEX6P1-RAMC were induced by IPTG for 4 hours. Lysate was prepared and incubated with glutathione-agarose beads. Beads were washed 4 times with the lysis buffer (50 mM Tris, pH 8.0, 125 mM NaCl, 1 mM EDTA, 1% Triton X-100, protease inhibitor cocktail (Sigma), twice with 50 mM Tris, pH 8.0, and then eluted with glutathione containing Tris buffer. Purified GST-RAMC protein was stored at −20°C.

### RT-PCR and Western Blot

RNA extraction and RT-PCR were performed according to standard protocols. PCR primers are: *Xenopus esr1*: 5′-ACAAGCAGGAACCCAATGTCA-3′ and 5′-GCCAGAGCTGATTGTTTGGAG-3′; zebrafish *odc*: 5′-CTGCTGTTCGAGAACATGGG-3′ and 5′-CTGCTACAGCACTTGAGTCG-3′; zebrafish *her6*: 5′- CAAATGACCGCTGCCCTAAAC-3′ and 5′-TGACTGAAGGATGGATGAGGAGG-3′; and zebrafish *her9*: 5′-CCAGCGTTTGCTTCTGCTACAAC-3′ and 5′-GCTCATTGCTTTCTGCTCCG-3′.

We used the NP-40 buffer (50 mM Tris, pH 8.0, 125 mM NaCl, 1 mM EDTA, 1% NP-40, protease inhibitor cocktail (Sigma) to extract proteins from embryos. Generally, 15 embryos were homogenized in 300 ml cold lysis buffer. Protein lysates were cleared by spinning the samples twice at 4°C. Subsequently, samples were separated on SDS-PAGE and analyzed by Western blotting as described [58]. Antibodies were anti-Myc (9E10, Sigma, 1∶1,000), anti-β-tubulin (mAb, Sigma, 1∶5,000), anti-GST (mAb, Santa Cruz, 1∶500), anti-GFP (mAb, Sigma, 1∶1,000), and HRP-linked donkey anti-mouse IgG (G&E, 1∶5,000).

## Supporting Information

Figure S1 NICD and Hes1 double immunostaining of the control and *brom bones* intestines.
(1.96 MB TIF)
