# Supplementary figures and images for: hnRNP I Inhibits Notch Signaling and Regulates Intestinal Epithelial Homeostasis in the Zebrafish
Source: PLoS Genet. 2009 Feb 6;5(2):e1000363. doi: 10.1371/journal.pgen.1000363 (PMC2629577; doi:10.1371/journal.pgen.1000363)

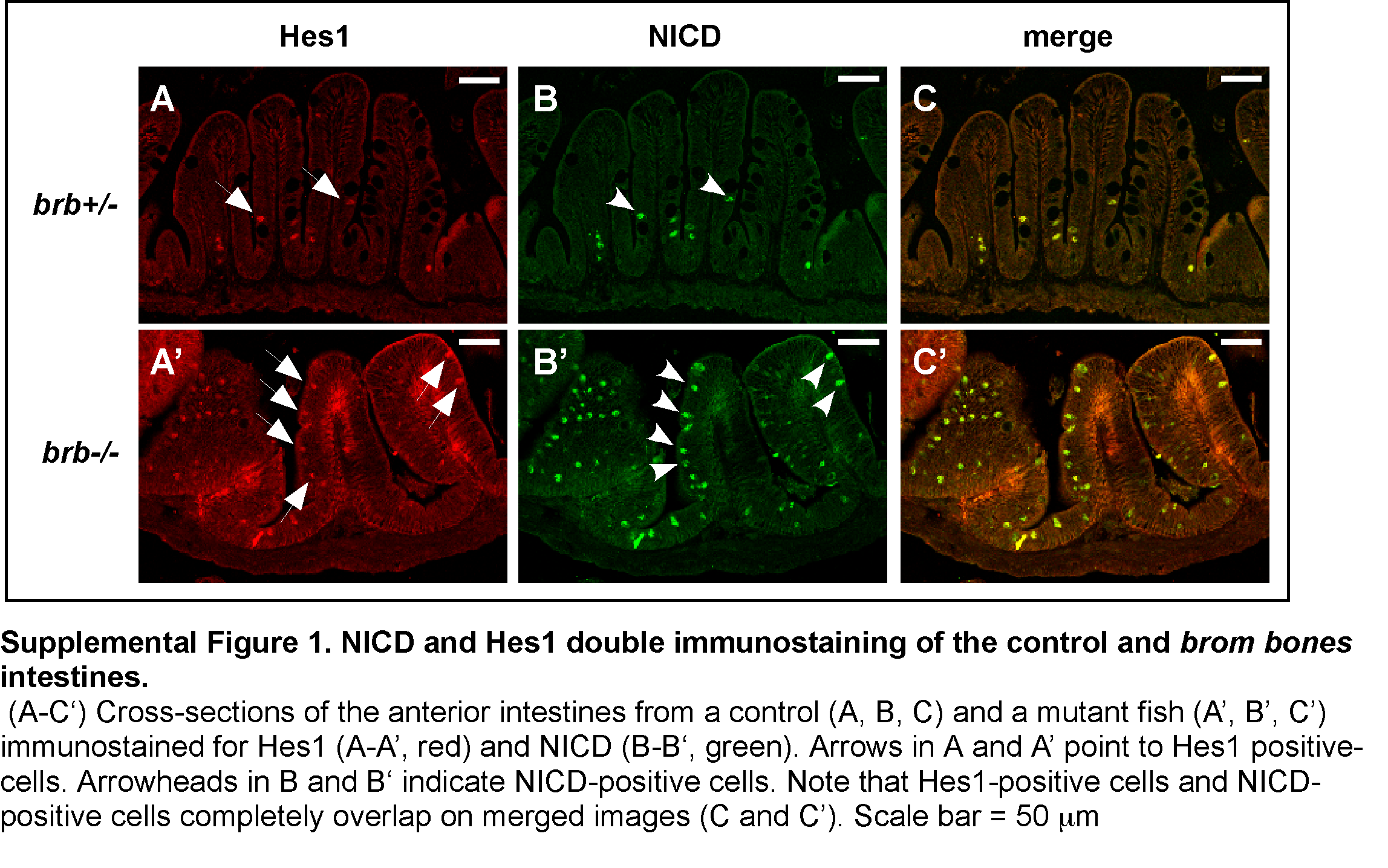

Supplement: Figure S1 — NICD and Hes1 double immunostaining of the control and brom bones intestines. (1.96 MB TIF) [file pgen.1000363.s001.tif]
